# New insights into earthquake precursors from InSAR

**DOI:** 10.1038/s41598-017-12058-3

**Published:** 2017-09-20

**Authors:** Marco Moro, Michele Saroli, Salvatore Stramondo, Christian Bignami, Matteo Albano, Emanuela Falcucci, Stefano Gori, Carlo Doglioni, Marco Polcari, Marco Tallini, Luca Macerola, Fabrizio Novali, Mario Costantini, Fabio Malvarosa, Urs Wegmüller

**Affiliations:** 1 Istituto Nazionale di Geofisica e Vulcanologia, Via di Vigna Murata 605, 00143 Roma, Italy; 2 Dipartimento di Ingegneria Civile e Meccanica (DICeM), Università degli Studi di Cassino e del Lazio meridionale, Via G. di Biasio 43, 03043 Cassino, Italy; 3 Dipartimento di Ingegneria Civile, Edile-Architettura e Ambientale, Università dell’Aquila, Via Giovanni Gronchi 18, 67100 L’Aquila, Italy; 4 TRE ALTAMIRA s.r.l., Ripa di Porta Ticinese, 20143 Milano, Italy; 5 e-GEOS Via Tiburtina, 965, 00156 Rome, Italy; 6 GAMMA Remote Sensing Research and Consulting AG, Worbstr. 225, CH-3073, Gümligen, Switzerland

## Abstract

We measured ground displacements before and after the 2009 L’Aquila earthquake using multi-temporal InSAR techniques to identify seismic precursor signals. We estimated the ground deformation and its temporal evolution by exploiting a large dataset of SAR imagery that spans seventy-two months before and sixteen months after the mainshock. These satellite data show that up to 15 mm of subsidence occurred beginning three years before the mainshock. This deformation occurred within two Quaternary basins that are located close to the epicentral area and are filled with sediments hosting multi-layer aquifers. After the earthquake, the same basins experienced up to 12 mm of uplift over approximately nine months. Before the earthquake, the rocks at depth dilated, and fractures opened. Consequently, fluids migrated into the dilated volume, thereby lowering the groundwater table in the carbonate hydrostructures and in the hydrologically connected multi-layer aquifers within the basins. This process caused the elastic consolidation of the fine-grained sediments within the basins, resulting in the detected subsidence. After the earthquake, the fractures closed, and the deep fluids were squeezed out. The pre-seismic ground displacements were then recovered because the groundwater table rose and natural recharge of the shallow multi-layer aquifers occurred, which caused the observed uplift.

## Introduction

The identification of earthquake precursors is a key area of investigation in modern seismology. Precursor signals have not been conclusively identified so far, even using modern geodetic techniques (e.g., GPS and SAR interferometry)^1–3^. However, the latest satellite missions and processing algorithms may represent progress toward this goal.

We present evidence of ground deformation preceding the 2009 L’Aquila earthquake. We observed this deformation using multi-temporal InSAR techniques, and we propose a plausible causative mechanism.

The April 6, 2009 M_w_ 6.3 L’Aquila earthquake occurred in central Italy. The event was generated by a 13-km-long, NW-striking and SW-dipping normal fault that borders the L’Aquila basin to the northeast^4–7^. Centroid-moment tensor solutions and the distribution of aftershocks define a fault plane that dips 45° between depths of 1 to 10 km^8,9^. We investigated the seismic cycle associated with the earthquake by applying advanced InSAR techniques to SAR datasets from various satellite missions (RADARSAT-2, Envisat and COSMO-SkyMed) that differ in terms of their wavelengths and spatial resolutions. Specifically, 159 RADARSAT-2 images (75 collected along descending orbits and 84 collected along ascending orbits) that span the six years preceding the mainshock were processed with the SqueeSAR^TM^ software package^10^, and 38 Envisat images collected along descending orbits that cover almost the same temporal interval as the RADARSAT-2 data were processed with the IPTA software package^11^. In addition, 35 COSMO-SkyMed images that were collected along ascending orbits and cover the 16 months following the earthquake were processed using the Persistent Scatterer Pair (PSP) technique^12^.

## Results

### InSAR results

**Figure 1:**
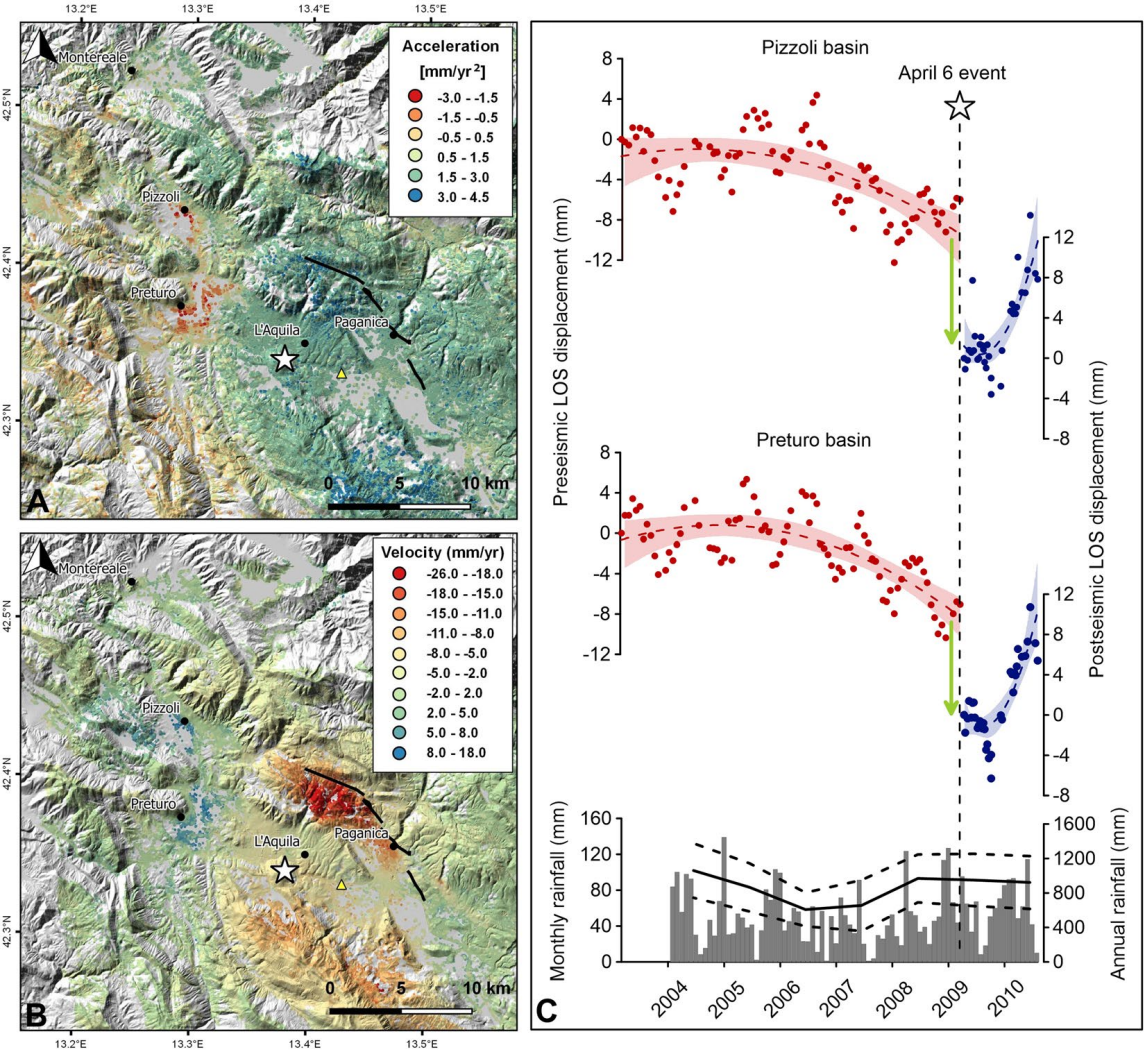
(**A**) Ground acceleration map extracted by multi-temporal InSAR processing of RADARSAT-2 data collected along ascending orbits. The map shows two distinct patterns of negative values (red and orange PSs) located within the Preturo and Pizzoli sedimentary basins. The corresponding time series of the averaged PSs are shown in panel C (red circles) for the Pizzoli and Preturo basins, respectively. (**B**) Post-seismic velocity map derived from COSMO-SkyMed data acquired along ascending orbits. Comparison of A and B shows that the Preturo and Pizzoli basins subsided from 2006 until the 2009 earthquake and experienced uplift during the post-seismic period. The uplift began in October-December 2009, as shown in panel C (blue circles). Red and blue dashed lines indicate a second-order polynomial fit. The red and blue bands indicate the 95% confidence interval. The white star indicates the epicenter of the April 6, 2009 earthquake on the maps and the timing of the earthquake in panel C. The gray vertical bars indicate the monthly rainfall at the L’Aquila – S. Elia pluviometric station (the yellow triangle in panel A and B). The black line indicates the annual rainfall calculated as the mean value of eight pluviometric stations (see Fig. 2 for the locations of the pluviometric stations), whereas the dashed black lines indicate the standard deviation with respect to the mean value. This figure was created with QGIS version 2.18.10 (QGIS Development Team, 2009. QGIS Geographic Information System. Open Source Geospatial Foundation. http://qgis.osgeo.org) and Grapher^®^ 8 from Golden Software, LLC (www.goldensoftware.com).

The RADARSAT-2 and Envisat mean velocity maps show very similar results during the pre-seismic phase; however, no significant spatial pattern of linear deformation trends was observed in these maps (Figure S1). To identify possible non-linear ground displacement over time, we computed maps of ground acceleration, which was calculated as double the quadratic coefficients of second-order polynomials fitted to the displacement time series of each persistent scatterer (PS). The computed acceleration maps (Figs 1A, S2A and B) reveal noteworthy variations in the deformation velocities measured using the data collected along both ascending and descending orbits. In particular, positive values were observed within the imaged area except in two regions, the Quaternary Preturo and Pizzoli basins. These basins, which are located on the northwestern rim of the coseismic deformation field (Figure S3), are characterized by generally negative values of up to −3 mm/yr^2^. Similar patterns can be observed in the data collected along both ascending and descending orbits, demonstrating the existence of predominantly vertical movement. We thus averaged the time series of the PSs within each basin in order to reduce oscillations and noise in the data and to obtain corresponding time series for the ascending and descending orbits. The ground deformation over time in both of the basins displayed comparable trends (Figs 1C and S2C), in addition to a yearly periodic signal. Slow uplift began in 2003 and was followed by significant subsidence after 2006. This subsidence, which reached mean values of approximately 1 cm, persisted until the earthquake.

In contrast, during the post-seismic phase, the COSMO-SkyMed ascending velocity map (Fig. 1B) shows subsidence associated with tectonic (afterslip) and gravitational phenomena^13,14^ over the entire imaged area (negative values), with the exception of the two basins mentioned above. The satellite data indicate progressive uplift within these basins that is characterized by velocities of approximately 5 to 18 mm/yr. This uplift began approximately 8 months after the mainshock and lasted at least 9 months (Fig. 1C).

**Figure 2:**
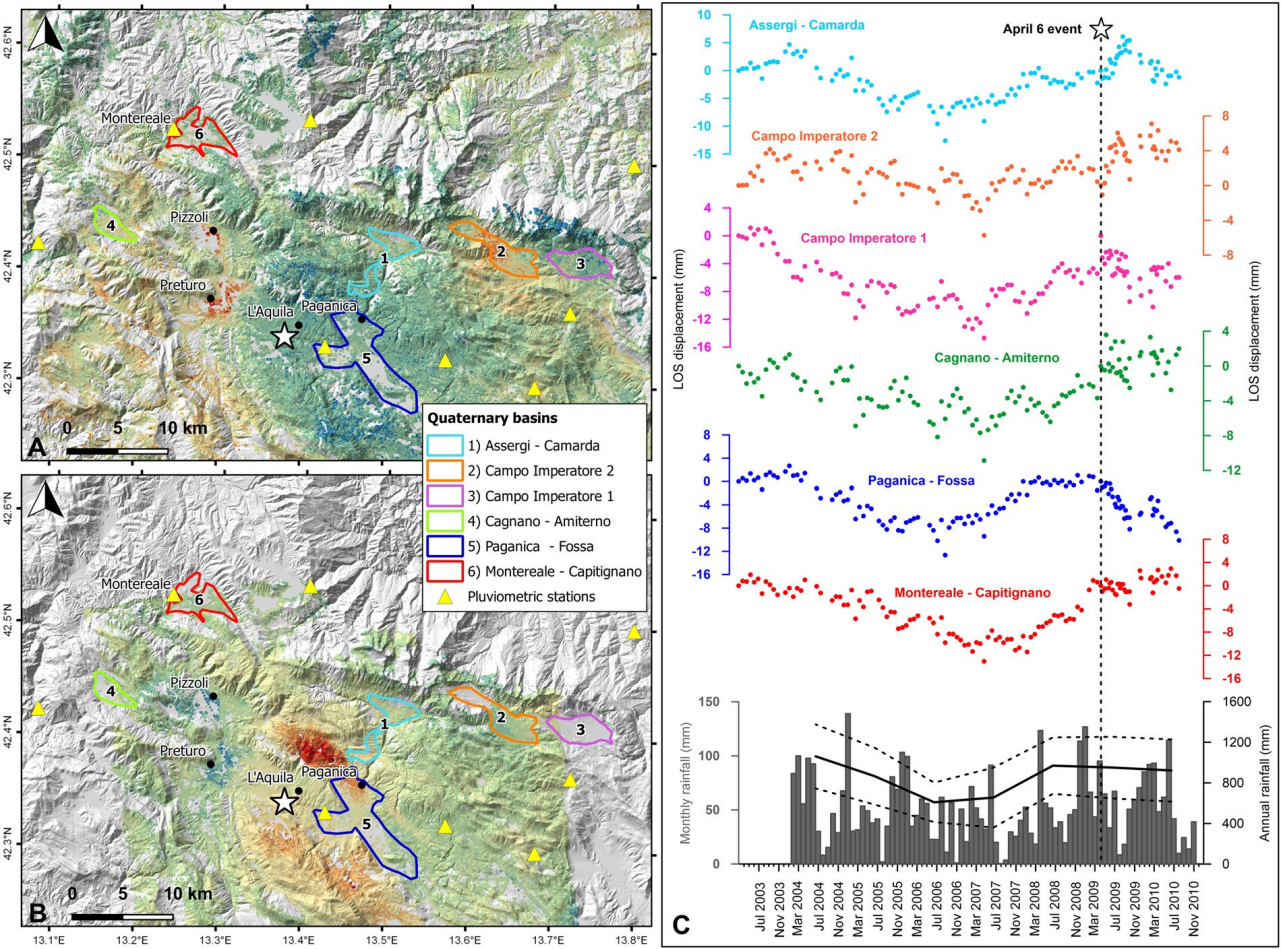
(**A**) Ground acceleration map extracted by multi-temporal InSAR processing of RADARSAT-2 data collected along ascending orbits (see Fig. 1A for the legend), (**B**) Post-seismic velocity map derived from COSMO-SkyMed data acquired along ascending orbits (see Fig. 1B for the legend). Both panels A and B show the locations of the Quaternary basins and the pluviometric stations in the vicinity of the Preturo and Pizzoli basins. (**C**) Pre- and post-seismic displacement time series of the averaged PSs falling within each of the Quaternary basins shown in panels A and B. The gray vertical bars indicate the monthly rainfall at the L’Aquila – S. Elia pluviometric station (the yellow triangle in Fig. 1A and B). The black line indicates the annual rainfall calculated as the mean value among the eight pluviometric stations shown in panels A and B, whereas the dashed black lines indicate the standard deviation with respect to the mean value. This figure was created with QGIS version 2.18.10 (QGIS Development Team, 2009. QGIS Geographic Information System. Open Source Geospatial Foundation. http://qgis.osgeo.org) and Grapher^®^ 8 from Golden Software, LLC (www.goldensoftware.com).

This analysis emphasizes that the Preturo and Pizzoli basins are the only two regions within the imaged area that subsided before the mainshock and then experienced uplift during the post-seismic phase (Fig. 1). Indeed, the neighboring Quaternary basins (Fig. 2A,B) show different patterns of displacement during the pre- and post-seismic phases. Namely, during the pre-seismic phase, the averaged time series of the PSs within each basin show very similar seasonal oscillations superimposed on multi-annual ground deformation (Fig. 2C). During the post-seismic phase, all of the neighboring basins show negligible ground deformation, except for the Paganica-Fossa basin, where ground subsidence associated with the afterslip^13^ occurs. The Paganica-Fossa basin is located in the hanging wall of the earthquake fault.

### Geological, hydrogeological and geotechnical setting

This section provides an outline of the geological, hydrogeological and geotechnical features of the Preturo and Pizzoli basins that supports the interpretation of the observed pre- and post-seismic deformation.

**Figure 3:**
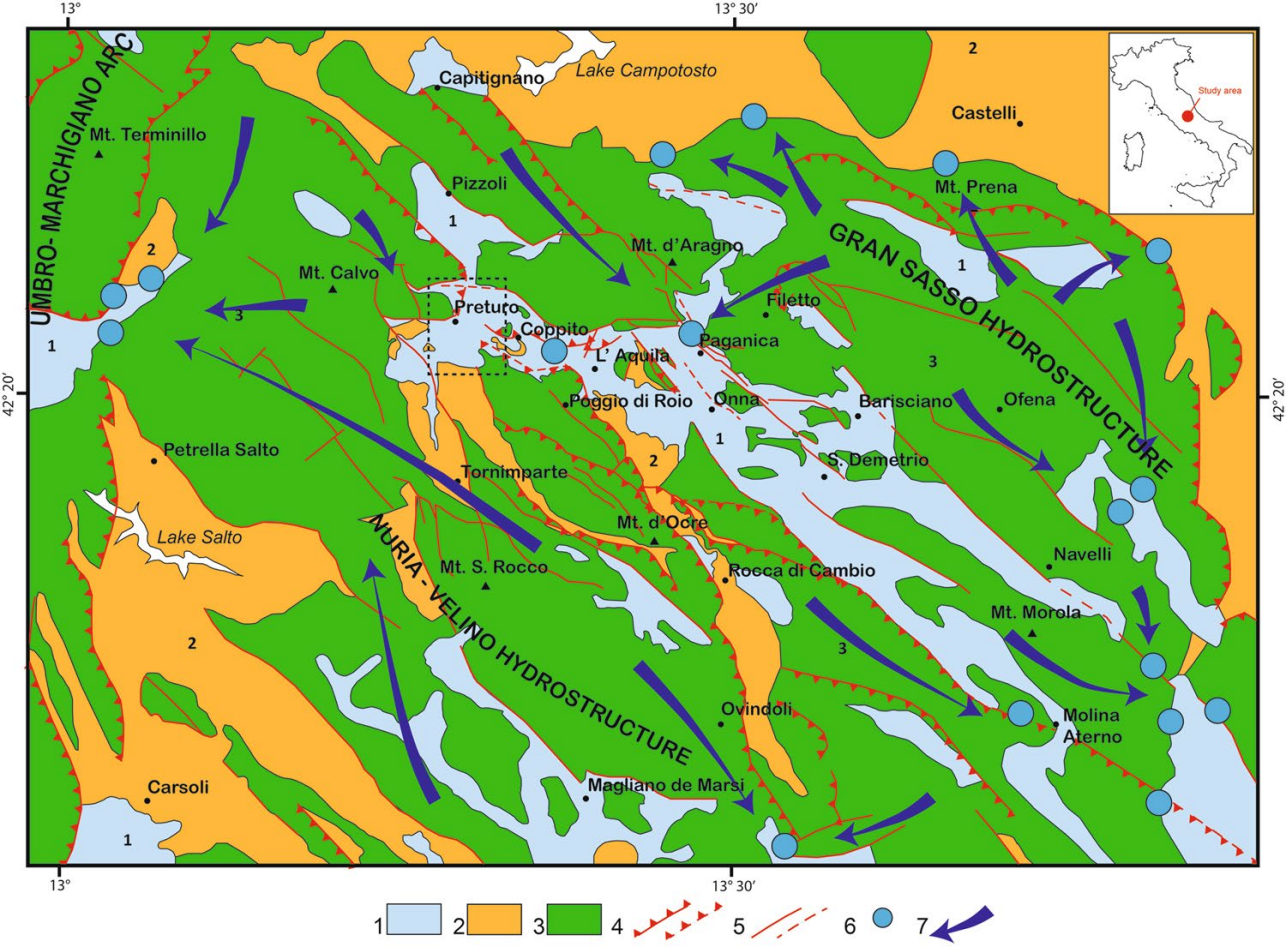
Geological and hydrogeological scheme of the study area. Black dashed rectangle indicates the area shown in Fig. 4A. Figure legend: 1) Quaternary alluvial and lacustrine complex (multi-layer aquifer). 2) Terrigenous complex (regional aquitard; Upper Miocene). 3) Upper Lias-Lower Miocene carbonate complex (aquifer). 4) Thrust fault (dashed where inferred). 5) Normal fault (dashed where inferred). 6) Major spring. 7) Groundwater flow path. The geological and hydrogeological scheme and the sketch of Italy were redrawn and modified form the “Schema Idrogeologico dell’Italia centrale” (Hydrogeological chart of Central Italy)^38^, freely available at: http://www.idrogeologiaquantitativa.it/?p=428&lang=it. This figure was created with Adobe^®^ Illustrator CS6 ver. 16 (http://www.adobe.com/products/illustrator.html).

**Figure 4:**
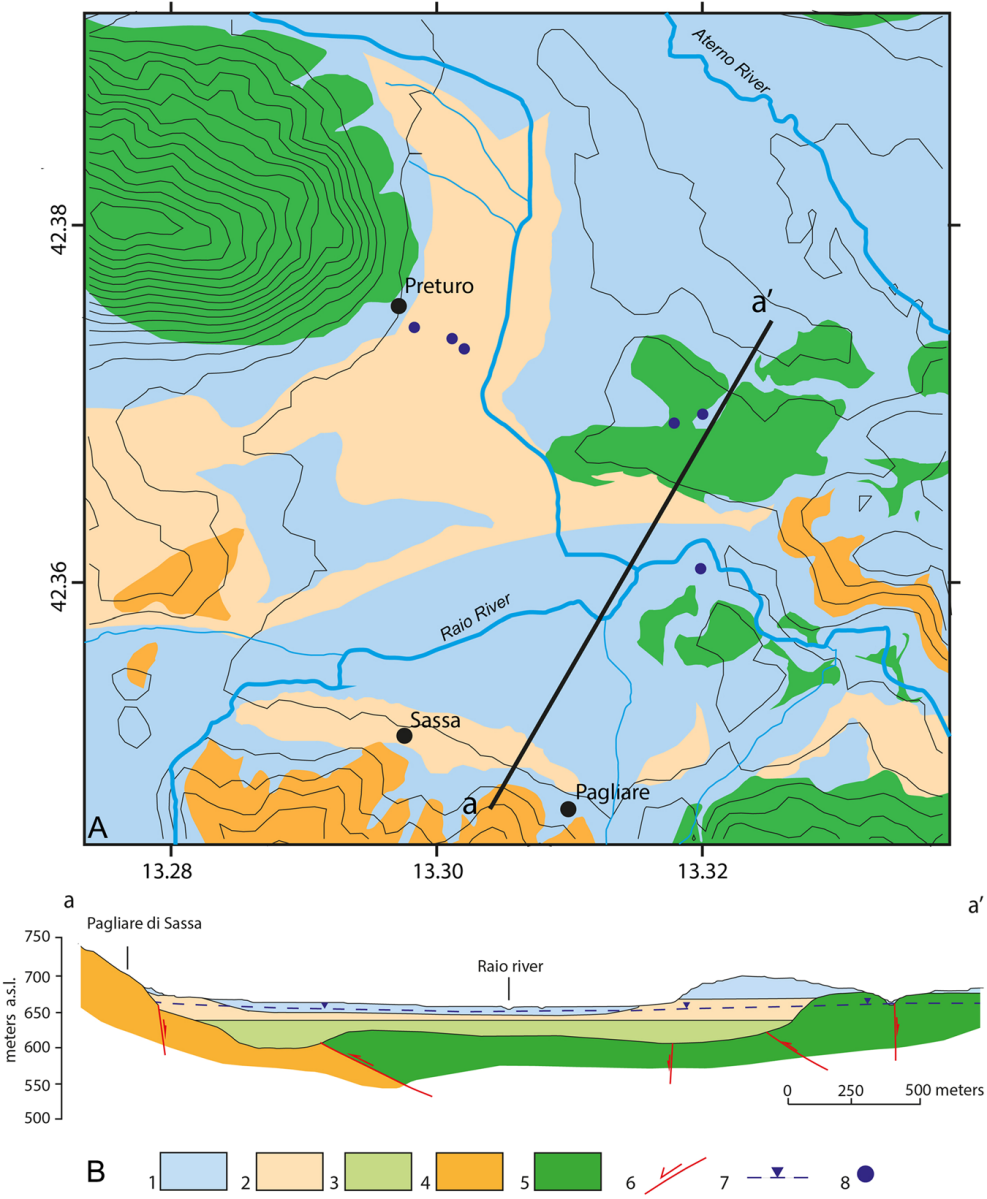
Simplified geological map (**A**) and cross section (**B**) of the Preturo basin. Figure legend: 1) Middle Pleistocene-Holocene alluvial and colluvial complex consisting of sand, gravel and silt (aquifer). 2) Lower Pleistocene lacustrine complex consisting of sand, silt and clay with lignite (aquitard/aquifer). 3) Upper Pliocene-Lower Pleistocene alluvial complex consisting of gravel, sand and silt (aquifer). 4) Upper Miocene sandstone and pelite complex (regional aquitard). 5) Upper Lias-Lower Miocene carbonate complex making up the Gran Sasso hydrogeological system (regional aquifer); 6) normal, contractional and/or strike-slip fault; 7) water table level; 8) borehole. Lithologies and soil thickness are from Nocentini *et al*.^16^. This figure was created with Adobe^®^ Illustrator CS6 ver. 16 (http://www.adobe.com/products/illustrator.html).

The Preturo and Pizzoli plains belong to the intramontane Quaternary basin of L’Aquila, the evolution of which is controlled by two active and SW-dipping normal faults, the Pettino Mt. and Marine Mt. faults. The plains are underlain by continental detrital deposits that unconformably overlie Meso-Cenozoic bedrock (Fig. 3). The latter rocks and the surrounding mountains consist primarily of carbonates, with some terrigenous Meso-Cenozoic lithologies. The Quaternary succession can be divided into three groups of synthems that vary in thickness^15,16^ (Fig. 4). Here, we describe these synthems from bottom to top. The basal material within the basins is composed of approximately 50–100 m of coarse-grained upper Piacenzian-Gelasian deposits (slope-derived breccia, debris flow and proximal alluvial fan deposits) that are rarely exposed (layer 3 in Fig. 4B). These deposits are overlain by approximately 50 m of Calabrian meandering fluvial-floodplain-swamp sediments that are characterized by sandy silt and sand beds interlayered with clay and clayey silts (layer 2 in Fig. 4B). Lignite intercalations also occur, and these intercalations become more frequent and thicker to the west. Layers of coarse to medium sand and gravel are present in the middle and lower parts of this sedimentary package. The third synthem consists of upper Pleistocene-Holocene braided fluvial floodplain deposits and slope-derived, clast-supported, medium to coarse gravel beds with primarily sandy matrix material (layer 1 in Fig. 4B).

The Preturo and Pizzoli plains are positioned between two major carbonate aquifers (the Gran Sasso aquifer to the north and the Nuria-Velino Mts. aquifer to the south and west) that differ in terms of their hydrogeologic relationships with the detrital multi-layer aquifers corresponding to the Quaternary basin-filling deposits^17^ (Fig. 3). The detrital multi-layer aquifers are fed by groundwater flowing laterally out of the Gran Sasso carbonate aquifer toward the center of the plains and by precipitation falling on the plains. Moreover, the presence of bedrock that is composed of low-permeability Miocene terrigenous and marly lithologies and underlies the multi-layer aquifers in the southern and western parts of the basins indicates that the Preturo and Pizzoli plains represent a minor secondary discharge area for the Nuria-Velino Mts. hydrostructure.

The Gran Sasso and Nuria-Velino Mts. carbonate aquifers are fissured and fault-partitioned media. Infiltration is very high (800 to 1200 mm/yr, which represents more than 60% of total precipitation)^18^. In contrast, runoff is negligible (approximately 5% of total precipitation). The movement of groundwater may be controlled by the presence of faults, which represent discontinuities with high or low hydraulic gradients and low- or high-permeability boundaries. Nevertheless, at the regional scale, the groundwater within the carbonate aquifers may be regarded as continuous. The substantial quantity of groundwater within the carbonate aquifers flows from the core of each aquifer to the borders, where the aquifers are drained by springs that have steady and high discharges (0.1–10 m^3^/s) (Fig. 3). The discharge of groundwater is controlled by the morphology and the altitude of the permeability boundary. The aquifer boundary coincides with the contact with the detrital fill underlying the two plains. The boundary is not totally free of discharge, and groundwater flows into the Quaternary multi-layer aquifer. These background conditions promote the recharge of the Quaternary multi-layer aquifer by the carbonate aquifers.

Regarding the hydrogeologic behavior of the Quaternary deposits, the Calabrian fine-grained sediment (layer 2 in Fig. 4B) corresponds to an aquitard that hydraulically confines or locally semi-confines the underlying upper Piacenzian-Gelasian coarse-grained deposit, whereas the overlying upper Pleistocene-Holocene coarse-grained deposit and the sandy layers at the top of the Calabrian succession correspond to an unconfined aquifer. In spite of this hydrogeologic setting, in which the Calabrian aquitard is enclosed between two aquifers (i.e., the upper Piacenzian-Gelasian deposit and the upper Pleistocene-Holocene coarse-grained deposit, which are found above and below the aquitard, respectively), the piezometric equilibrium of the Quaternary multi-layer aquifer is based on a single water table. Thus, an upward component of groundwater flow into the Calabrian aquitard that occasionally displays artesian behavior cannot be excluded.

Earthquake-induced paleohydrogeological phenomena (sinkholes and liquefaction features) have occurred in the two plains during the middle Pleistocene and in historical times. Together with modern piezometric data, these observations confirm the hydrogeological background described above^19^.

The main geotechnical properties of the Quaternary sedimentary soils are derived from a series of *in situ* surveys performed in the Preturo basin (Fig. 4A) and laboratory testing^20^. The typical stratigraphy of the area determined from borehole logs (Fig. 4B) shows a sequence of coarse- and fine-grained materials with strata of variable thickness. The results of laboratory tests on undisturbed fine-grained samples taken from the available boreholes show that this material consists primarily of silts (Figure S4A) (ranging between 20% and 80%), with a slightly lower content of clays (between 20% to 50%) and a small percentage of sand (between 0% and 20%). The consistency limits (i.e., the plasticity index, PI, and the liquid limit, LL) plotted on a Casagrande chart (Figure S4B) show that these sediments have a homogenous mineralogical composition and that the clay component exhibits medium to high plasticity. The compressibility and swelling indexes (denoted with *C*_*C*_ and *C*_*s*_, respectively, in Figure S5C and D) obtained from oedometer tests do not show any significant dependence with depth. Overconsolidation caused by seasonal fluctuations in the water table is present in the upper meters of the basin fill; in Figure S5B, the overconsolidation ratio (OCR) ≫1.

Based on the available results, the fine-grained soils are highly susceptible to consolidation in cases in which the stress field is modified, leading to ground subsidence.

## Discussion

Based on the observed InSAR ground deformations and on the regional and local geological, geotechnical and hydrogeologic features, we hypothesize that the most plausible explanation for the observed pre-seismic subsidence and post-seismic uplift is the consolidation and swelling of the fine-grained content of the alluvial deposits that fill the Preturo and Pizzoli basins.

**Figure 5:**
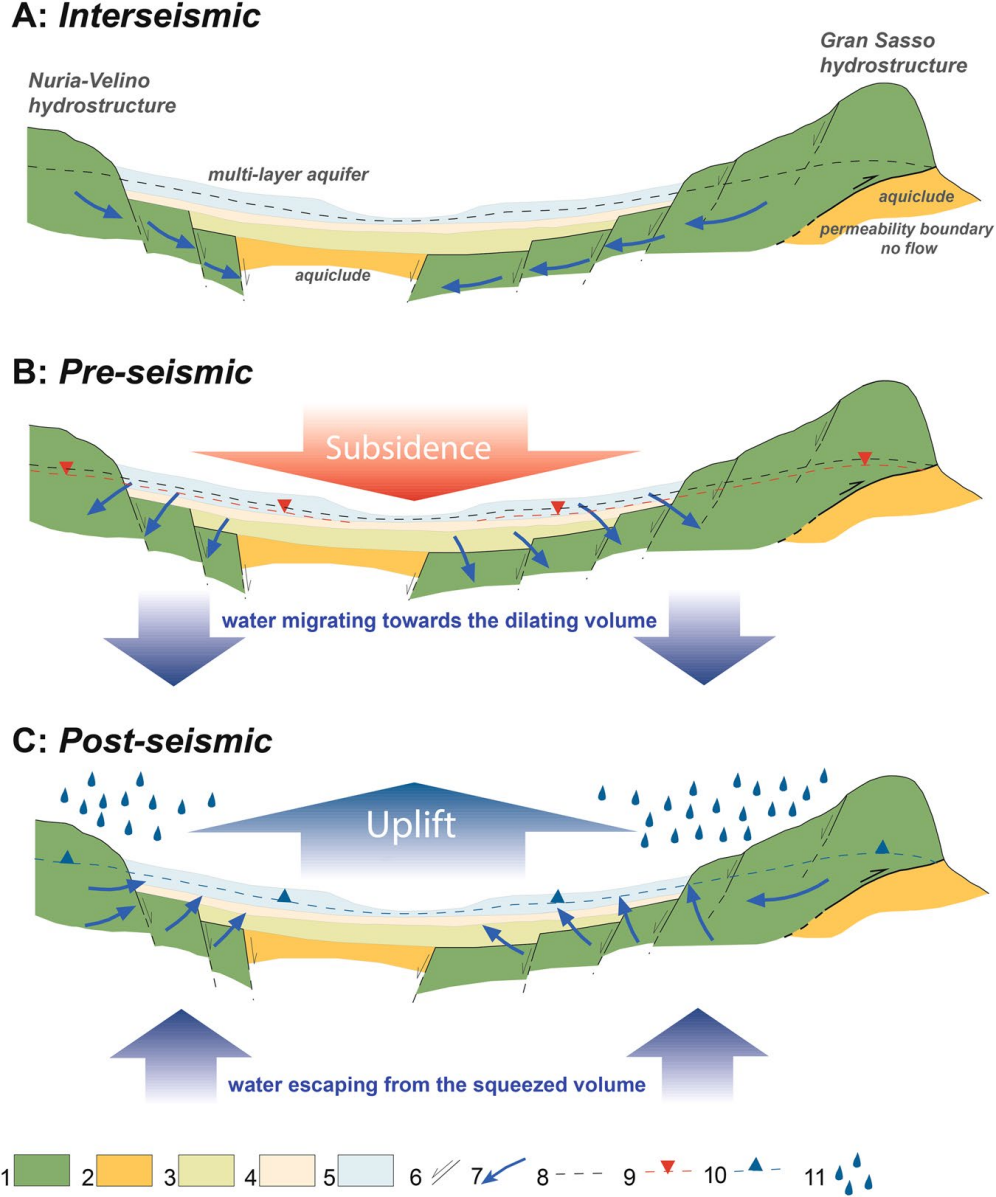
Simplified scheme describing the mechanism of the observed water table variations based on dilatancy theory. (**A**) Interseismic stage before the beginning of the dilatancy phase. (**B**) Pre-seismic phase: dilatancy occurs, causing the migration of water toward the dilating volume and producing lowering of the water table. This process results in the ground subsidence detected in the InSAR data. (**C**) Post-seismic phase: the fractures in the focal volume close and the water is squeezed out, refilling the carbonate aquifer and the overlying shallow multi-layer aquifer. As a consequence, the ground experiences uplift. Figure legend: 1) Jurassic-Lower Miocene carbonate complex. 2) Upper Miocene terrigenous complex. 3) Upper Pliocene-Lower Pleistocene gravel, sand and silt (aquifer). 4) Lower Pleistocene sand, silt and clay with lignite (aquitard/aquifer). 5) Middle Pleistocene-Holocene sand, gravel and silt (aquifer). 6) Fault. 7) Groundwater flow path. 8) Water Table. 9) Lowering of the water table during the pre-seismic phase. 10) Water table recovery during the post-seismic phase. 11) Recharge of the aquifer due to precipitation. This figure was created with Adobe^®^ Illustrator CS6 ver. 16 (http://www.adobe.com/products/illustrator.html).

The consolidation would have been caused by the long-term (i.e., not seasonal) lowering of the water table in the multi-layer aquifers in the basins. The contemporaneous, spatially homogeneous subsidence of the two basins is related to a decrease in the aquifer in the Gran Sasso hydrostructure. Indeed, these aquifers are hydrologically connected to the major hydrostructure of the Gran Sasso carbonate range (Fig. 5A); hence, large variations in the former are controlled by large variations in the latter. A lowering of groundwater levels in the Gran Sasso carbonate range was observed before the L’Aquila earthquake^21,22^. This decrease induced the migration of water from the connected multi-layer aquifers in the basins, which led to the observed subsidence (Fig. 5B).

On the other hand, the post-seismic uplift of the basins was induced by rising groundwater levels and the consequent recovery of the elastic portion of the ground settlement (Fig. 5C). Indeed, post-seismic increases in groundwater levels in the Gran Sasso aquifer have also been measured^22–25^.

Dilatancy theory accounts for the described process of water table variations^23,26–30^. It implies the formation of cracks due to shearing. Consequently, water diffuses through the rocks within the earthquake source volume (i.e., the focal volume).

In particular, during the pre-seismic phase, shear stresses were applied to the rocks in the focal volume, causing fractures and voids to open and the volume to increase. Hence, water migrated into the focal volume from the surroundings (i.e., the Gran Sasso aquifer), thus lowering the groundwater in the hydrologically connected multi-layer aquifer within the basins. Indeed, a complex sequence of dilatancy and the diffusion of fluid toward the earthquake nucleation area has been inferred from V_p_/V_s_ variations that began in October 2008^31,32^. Moreover, according to Stacey^33^, a three-year period represents a plausible preparatory phase for an earthquake with a magnitude comparable to the 2009 event.

During the post-seismic phase, the increase in groundwater levels likely resulted from both seasonal recharge of the aquifers (precipitation) and squeezing of the deep groundwater caused by coseismic dislocation, which led to the closure of fractures and voids and the expulsion of water from the focal volume^34,35^.

Although other phenomena can explain the water table oscillations in the two Quaternary basins, no significant industrial or agricultural activities occur in these basins. Thus, anthropogenically driven changes in the groundwater level can be neglected.

The effects of seasonal or multi-annual rainfall variations can be ruled out as well. In fact, we analyzed seasonal and multi-annual precipitation records collected at eight pluviometric stations located within the study area between 2004 and 2010 (Fig. 2A,B). A comparison of rainfall and ground deformation shows that the ground deformation within the Quaternary basins in the vicinity of Preturo and Pizzoli is clearly correlated with both seasonal and multi-annual rainfall variations (Fig. 2C). Conversely, the ground deformations that occurred within the Preturo and Pizzoli basins display no correlation with any of the multi-annual rainfall variations, only the seasonal ones (Fig. 1C).

Finally, we evaluated the consolidation potential of the fine-grained soils of the Preturo and Pizzoli basins in accordance with the classical principles of Terzaghi^36^, and we compared the numerical results with the InSAR observations.

The key factors affecting the consolidation of soils are the lowering of the water table, which reduces the pore water pressure and increases the overburden effective stress, and the compressibility of the soils at different depths. The magnitude and distribution of subsidence over the studied area should thus reflect the combined action of these two factors.

**Figure 6:**
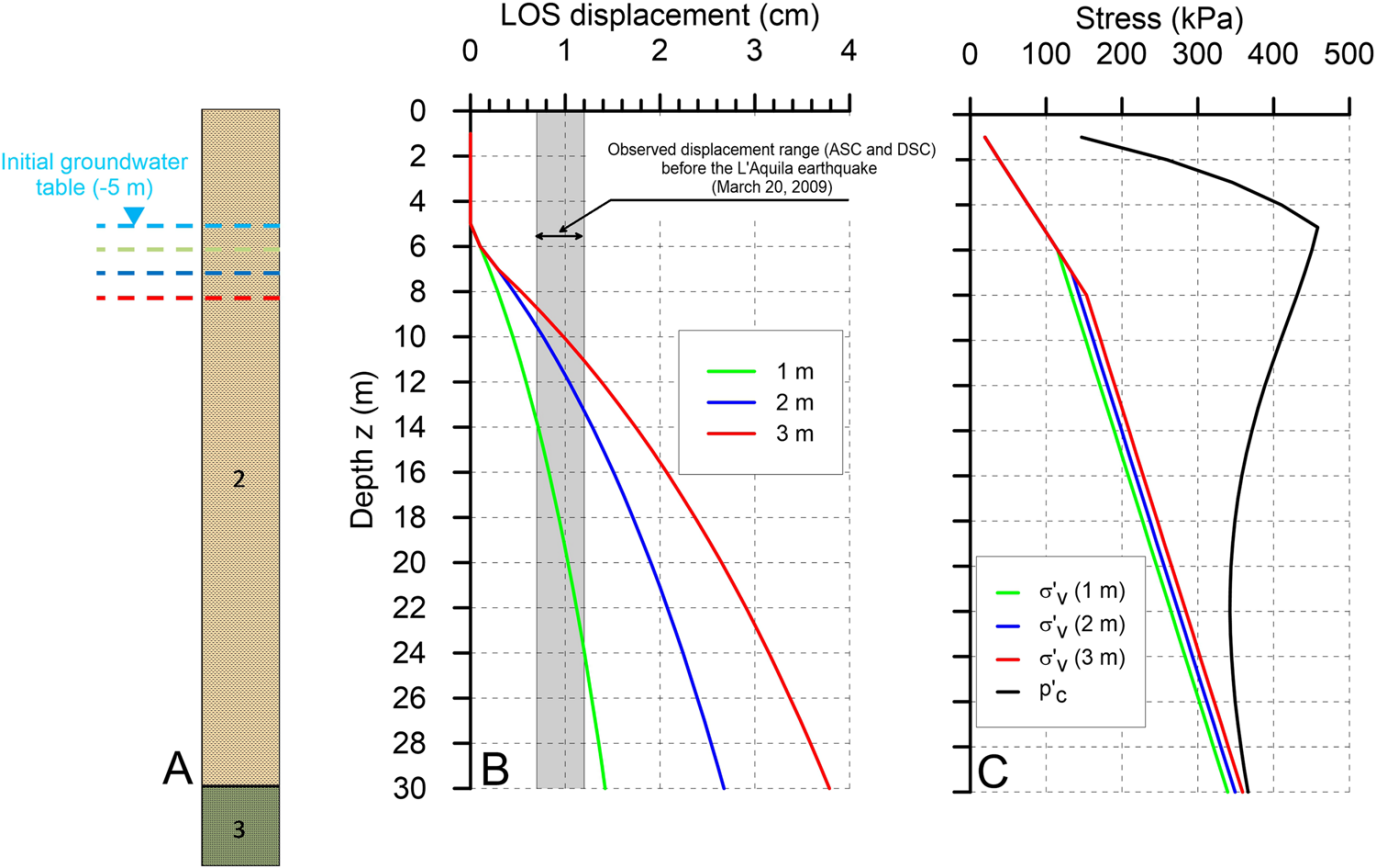
Simplified stratigraphy adopted for the consolidation analysis (**A**) and the results of the consolidation analysis. The initial groundwater table is fixed at a depth of −5 meters. The consolidation analysis is performed assuming groundwater level drops of 1 m (green line), 2 m (blue line) and 3 m (red line). (**B**) Amounts of ground settlement projected along the ascending (ASC) and descending (DSC) line of sight of the RADARSAT-2 satellites, computed assuming variable soil thicknesses (z) and groundwater table drops (1, 2, or 3 m). The gray band indicates the InSAR cumulated ground displacement range on the Preturo and Pizzoli basins before the L’Aquila earthquake. (**C**) Comparison of the preconsolidation stress (${p}_{C}^{^{\prime} }$) with the vertical effective stresses (${\sigma }_{v}^{^{\prime} }$) computed assuming variable groundwater table drops. This figure was created with Grapher^®^ 8 from Golden Software, LLC (www.goldensoftware.com).

The analysis is made with reference to the simplified 1D soil profile shown in Fig. 6A. The undisturbed water table level is assumed to be equal to the mean value measured in the available boreholes (approximately 5 meters below the ground level), and the water table drop from the undisturbed condition is varied between 1 and 3 meters. In this calculation, the compressibility and swelling indexes (*C*_*C*_ and *C*_*s*_), the natural unit weight (*γ*) and the initial void index (*e*_0_) have been assigned constant values that are equal to the means of the experimental values shown in Figure S5 (i.e., *γ* = 19.16 kN/m^3^, *C*_*C*_ = 0.267, *C*_*S*_ = 0.0578, and *e*_0_ = 0.785). The OCR is assumed to vary with depth according to the empirically derived power law shown in Figure S5B.

The results of the consolidation analysis, which is conducted for different depths (z) and water table drops (1–3 m), are shown in Fig. 6B. The computed settlement values are projected along the line of sight of the RADARSAT-2 satellites to enable comparison with the observations.

The results show that the cumulative InSAR pre-seismic ground displacement range at the time of the April 6 event (gray band in Fig. 6B) is well reproduced by the model when a lowering of the water table between 1 and 3 meters is assumed. The thickness of the fine-grained soils must lie between 10 and 25 meters, in agreement with the retrieved maximum and minimum thicknesses of the fine-grained deposits filling the two Quaternary basins. The similarity between the observed and simulated ground displacements provides a logical explanation for the observed subsidence, which can be considered to be the result of the delayed groundwater flow induced by dilatancy at depth, which caused a drop in the groundwater table. Moreover, the results of the consolidation analysis show that the computed displacements are elastic because the computed effective stress increase caused by the groundwater table lowering is always lower than the preconsolidation stress (Fig. 6C). Consequently, the computed ground displacements are recoverable if the groundwater table rises to its initial level (see the Methods section for details), thereby explaining the amplitude of the uplift measured during the post-seismic phase using the COSMO-SkyMed data (Fig. 1B,C).

In conclusion, this study provides an interpretation of the surface deformation preceding the L’Aquila earthquake in terms of seismic precursors. The InSAR-derived ground accelerations reveal signals that may not be evident in standard mean velocity maps. In seismogenic areas characterized by the presence of Quaternary deposits and favorable hydrogeological settings (i.e., hydraulically connected shallow and deep aquifers), ground acceleration maps can be powerful tools for predicting earthquakes.

Although this study investigates only one earthquake, we believe that it provides a new methodology that can be applied in studies of earthquake precursors and is novel in terms of the technology used and the signals that can be investigated.

## Methods

### InSAR data and analysis

We used several SAR datasets acquired by the COSMO-SkyMed (CSK), RADARSAT-2 (RS2) and Envisat missions.

The pre-seismic sequence was investigated using RS2 data captured along ascending and descending orbits and Envisat data captured along descending orbits. In contrast, the post-seismic sequence was investigated using CSK data.

The RS2 ascending dataset extends from March 4, 2003 to March 3, 2009. The SAR images were acquired at an incidence angle of 33° and an azimuth of 10°. The RS2 descending dataset extends from April 15, 2003 to March 14, 2009. This dataset was processed with the SqueeSAR technique^10^ and includes 84 and 75 SAR images collected along ascending and descending orbits, respectively.

The Envisat data, which were acquired along descending orbits, includes a stack of 50 images collected between November 10, 2002 and March 8, 2009. The line-of-sight direction is defined by a 24.5° incidence angle measured relative to the vertical direction and an aspect angle of −12.2 from the east. The dataset was processed with GAMMA IPTA software^11^ using a single reference stack.

The CSK dataset was acquired between April 12, 2009 and August 6, 2010 along ascending orbits with an incidence angle of 40°. This dataset consists of 35 SAR images covering an area of approximately 40 × 40 km^2^. The data were processed with the persistent scatterer pair (PSP) technique^12^, which permits estimation of displacement time series for dense sets of locations that correspond to identified persistent scatterers.

The obtained results present a standard deviation of the velocities between 0.6–0.9 mm/year. On the other hand, the standard deviation of the deformation measurement error is on the order of 2 mm and typically ranges between 1 and 3 mm, depending on the quality of the specific PS and the type of ground cover.

### Consolidation analysis

In the present study, the long-term settlement *w* caused by groundwater lowering was calculated assuming simplified 1D conditions (equation 1), in accordance with the classical principles of Terzaghi^36^:

1 $$w(z)={\int }_{H}{\varepsilon }_{z}\,\cdot \,dz$$

where H represents the thickness of the compacting layer, and *ε*_*z*_ is the vertical strain produced at each depth z by the increase in the effective overburden stress caused by the groundwater head drop. We assumed that the compressibility of the coarse-grained materials is negligible. This assumption was made because the compressibility indexes of silty–clayey and gravelly–sandy soils typically differ by some orders of magnitude. Indeed, whereas the oedometer tests performed on the fine-grained materials yielded compressibility index values ranging between 0.2 and 0.4 (*C*_*C*_ in Figure S5), values that are lower by some orders of magnitude are typically found for gravelly soils^37^.

Equation 1 implies that horizontal strains are negligible, i.e., the soil deforms under oedometric conditions (${\varepsilon }_{x}={\varepsilon }_{y}=0$ in Figure S6). This assumption is acceptable if we consider the almost flat geometry of the Preturo and Pizzoli basins (Fig. 4b) and a uniform lowering of the water table. Under these hypotheses, the resulting stress path corresponds to the red curve in Figure S6.

Discretizing the compacting layer into *n* sublayers with thickness Δ_*z*_, equation 1 simplifies to the following:

2 $$w(z)=\sum _{i=1}^{n}\frac{{\rm{\Delta }}{z}_{i}}{1+{e}_{0}}\cdot {\rm{\Delta }}{e}_{i}$$

where Δ_*zi*_ and ${e}_{0}$ are the thickness and the initial void index of the i^th^ layer, respectively, whereas Δ_*ei*_ is the change in the void index of the i^th^ layer and is given by the following:

3 $${\rm{\Delta }}{e}_{i}=\{\begin{array}{c}{C}_{c}\cdot {\mathrm{log}}_{10}(\frac{\sigma ^{\prime} {v}_{0,i}+{\rm{\Delta }}\sigma ^{\prime} v}{{\sigma }^{\text{'}}{v}_{0,i}})\,\,\,\,\,\,\,\,\,\,\,\,\,\,\,\,\,\,\,\,\,\,\,\,\,\,\,if\,\sigma ^{\prime} {v}_{0,i} > \,p^{\prime} {c}_{i}\,\,\,\,\,\\ {C}_{s}\cdot {\mathrm{log}}_{10}(\frac{\sigma ^{\prime} {v}_{0,i}+{\rm{\Delta }}\sigma ^{\prime} v}{\sigma ^{\prime} {v}_{0,i}})\,\,\,\,\,\,\,\,\,\,\,\,\,\,\,\,\,\,\,\,\,\,\,\,\,\,\,if\,\sigma ^{\prime} {v}_{0,i}+{\rm{\Delta }}\sigma ^{\prime} v < \,p^{\prime} {c}_{i}\\ {C}_{s}\cdot {\mathrm{log}}_{10}(\frac{p^{\prime} {c}_{i}}{\sigma ^{\prime} {v}_{0,i}})+{C}_{c}\cdot {\mathrm{log}}_{10}(\frac{\sigma ^{\prime} {v}_{0,i}+{\rm{\Delta }}\sigma ^{\prime} v}{p^{\prime} {c}_{i}})\,\,\,\,\,\,\,if\,\sigma ^{\prime} {v}_{0,i} < \,p^{\prime} {c}_{i} < \,\sigma ^{\prime} {v}_{0,i}+{\rm{\Delta }}\sigma ^{\prime} v\end{array}$$

In equation 3, *C*_*C*_ and *C*_*S*_ are the compressibility and swelling indexes, respectively; $\sigma ^{\prime} {v}_{0,i}$ is the initial effective vertical stress; ${\rm{\Delta }}\sigma ^{\prime} {v}_{i}$ is the effective vertical stress increase; and ${p}_{c,i}^{^{\prime} }$ is the preconsolidation stress, which is given by the following equation (4):

4 $${p}_{c,i}^{{\rm{^{\prime} }}}=OCR\cdot \,{\sigma }^{{\rm{^{\prime} }}}{v}_{0,i}$$

where OCR is the overconsolidation ratio (Figure S5B).

The vertical displacements computed using equations 2 and 3 may or may not be elastic (i.e., recoverable), depending on the relative positions of $\sigma ^{\prime} {v}_{0,i}$, ${p}_{c,i}^{^{\prime} }$ and ${\rm{\Delta }}\sigma ^{\prime} {v}_{i}$ on the oedometric curve. Initially, the soil has a stress state that is characterized by particular values of the effective vertical stress $\sigma ^{\prime} {v}_{0,i}$ and the void index ${e}_{0}$ (point a in Figure S6). An increase in the vertical effective stress ${\rm{\Delta }}\sigma ^{\prime} {v}_{i}$ causes the point to move to the right along the stress path (red line in Figure S6). However, if the initial vertical stresses exceed the preconsolidation stress ($\sigma ^{\prime} {v}_{0,i} > {p}_{c,i}^{^{\prime} }$ at point a’), the point moves along a stress path with a slope equal to *C*_*C*_, i.e., the virgin curve (Figure S6), thus producing elastic (recoverable) and plastic (unrecoverable) deformations. In contrast, if the initial vertical stress is lower than the preconsolidation stress $\sigma ^{\prime} {v}_{0,i} < {p}_{c,i}^{^{\prime} }$ at point a), the point moves along a stress path with a slope equal to *C*_*S*_, i.e., the swelling curve (Figure S6). In such cases, if the increase in the effective stress does not overcome the preconsolidation stress ($\sigma ^{\prime} {v}_{0,i}+{\rm{\Delta }}\sigma ^{\prime} {v}_{i}\, < {p}_{c,i}^{^{\prime} }$ at point b), the resulting strains are elastic (recoverable). On the other hand, if the increase in effective stresses overcomes the preconsolidation stress ($\sigma ^{\prime} {v}_{0,i}+{\rm{\Delta }}\sigma ^{\prime} {v}_{i} > {p}_{c,i}^{^{\prime} }$ at point c), the resulting strains will be partially unrecoverable.

## Electronic supplementary material

Supplementary figures

## References

[1] Arimoto, M., Fukushima, Y., Hirahara, K. & Hashimoto, M. An attempt to detect preseismic displacement field of the 2008 Iwate-Miyagi Nairiku Earthquake using InSAR small baseline time-series analysis. in *AGU Fall Meeting Abstracts* **1**, 596 (2008).

[2] Raucoules D, Ristori B, de Michele M, Briole P (2010). Surface displacement of the Mw 7 Machaze earthquake (Mozambique): Complementary use of multiband InSAR and radar amplitude image correlation with elastic modelling. Remote Sens. Environ..

[3] Atzori S, Chiarabba C, Devoti R, Bonano M, Lanari R (2013). Anomalous far-field geodetic signature related to the 2009 L’Aquila (central Italy) earthquake. Terra Nov..

[4] Falcucci E (2009). The Paganica Fault and Surface Coseismic Ruptures Caused by the 6 April 2009 Earthquake (L’Aquila, Central Italy). Seismol. Res. Lett..

[5] Boncio, P. *et al*. Coseismic ground deformation of the 6 April 2009 L’Aquila earthquake (central Italy, Mw 6.3). *Geophys. Res. Lett*. **37** (2010).

[6] Galli P, Giaccio B, Messina P (2010). The 2009 central Italy earthquake seen through 0.5 Myr-long tectonic history of the L’Aquila faults system. Quat. Sci. Rev..

[7] Moro M (2013). Historical earthquakes and variable kinematic behaviour of the 2009 L’Aquila seismic event (central Italy) causative fault, revealed by paleoseismological investigations. Tectonophysics.

[8] Chiarabba C (2009). The 2009 L’Aquila (central Italy) Mw 6.3 earthquake: Main shock and aftershocks. Geophys. Res. Lett..

[9] Valoroso L (2013). Radiography of a normal fault system by 64,000 high-precision earthquake locations: The 2009 L’Aquila (central Italy) case study. J. Geophys. Res. Solid Earth.

[10] Ferretti A (2011). A New Algorithm for Processing Interferometric Data-Stacks: SqueeSAR. IEEE Trans. Geosci. Remote Sens..

[11] Werner, C., Wegmuller, U., Strozzi, T. & Wiesmann, A. Interferometric point target analysis for deformation mapping. In *IEEE International Geoscience and Remote Sensing Symposium. Proceedings* (*IEEE Cat. No. 03CH37477*) **7**, 4362–4364 (IEEE, 2003).

[12] Costantini, M., Falco, S., Malvarosa, F. & Minati, F. A New Method for Identification and Analysis of Persistent Scatterers in Series of SAR Images. In *IGARSS 2008-2008 IEEE International Geoscience and Remote Sensing Symposium* II-449-II-452. 10.1109/IGARSS.2008.4779025 (IEEE, 2008).

[13] D’Agostino N (2012). Space-time distribution of afterslip following the 2009 L’Aquila earthquake. J. Geophys. Res..

[14] Albano M (2015). Gravity-driven postseismic deformation following the Mw 6.3 2009 L’Aquila (Italy) earthquake. Sci. Rep..

[15] Cosentino, D. *et al*. New insights into the onset and subsequent evolution of the central Apennine extensional intermontane basins from the tectonically active L’Aquila Basin (central Italy). *GSA Bull*. 10.1130/B31679.1 (2017).

[16] Nocentini M (2017). Plio-Quaternary geology of L’Aquila – Scoppito Basin (Central Italy). J. Maps.

[17] Petitta M, Tallini M (2003). Groundwater resources and human impacts in a Quaternary intramontane basin (L’Aquila Plain, Central Italy). Water Int..

[18] Petitta M, Tallini M (2002). Idrodinamica sotterranea del massiccio del Gran Sasso (Abruzzo): nuove indagini idrologiche, idrogeologiche e idrochimiche (1994-2001). Boll. della Soc. Geol. Ital..

[19] Storti F (2013). Evidence for strong middle Pleistocene earthquakes in the epicentral area of the 6 April 2009 L’Aquila seismic event from sediment paleofluidization and overconsolidation. J. Geophys. Res. Solid Earth.

[20] Monaco, P. *et al*. Geotechnical Aspects of the L’Aquila Earthquake. *Geotech. Geol. Earthq. Eng*. 1–66, 10.1007/978-94-007-2060-2_1 (2012).

[21] Amoruso A, Crescentini L, Martino S, Petitta M, Tallini M (2014). Correlation between groundwater flow and deformation in the fractured carbonate Gran Sasso aquifer (INFN underground laboratories, central Italy). Water Resour. Res..

[22] Amoruso A, Crescentini L, Petitta M, Rusi S, Tallini M (2011). Impact of the 6 April 2009 L’Aquila earthquake on groundwater flow in the Gran Sasso carbonate aquifer, Central Italy. Hydrol. Process..

[23] Muir-Wood R, King GCP (1993). Hydrological signatures of earthquake strain. J. Geophys. Res. Solid Earth.

[24] Adinolfi Falcone R (2012). Changes on groundwater flow and hydrochemistry of the Gran Sasso carbonate aquifer after 2009 L’Aquila earthquake. Ital. J. Geosci..

[25] Amoruso A, Crescentini L, Petitta M, Tallini M (2013). Parsimonious recharge/discharge modeling in carbonate fractured aquifers: The groundwater flow in the Gran Sasso aquifer (Central Italy). J. Hydrol..

[26] Nur A (1972). Dilatancy, pore fluids, and premonitory variations of ts/tp travel times. Bull. Seismol. Soc. Am..

[27] Aggarval YP, Sykes LR, Armbruster J, Sbar ML (1973). Premonitory Changes in Seismic Velocities and Prediction of Earthquakes. Nature.

[28] Scholz CH, Sykes LR, Aggarwal YP (1973). Earthquake Prediction: A Physical Basis. Science (80-.)..

[29] Nur A (1974). Matsushiro, Japan, Earthquake Swarm: Confirmation of the Dilatancy-Fluid Diffusion Model. Geology.

[30] Manga M, Wang C-Y (2015). Earthquake Hydrology. Treatise Geophys..

[31] Lucente FP (2010). Temporal variation of seismic velocity and anisotropy before the 2009 Mw 6.3 L’Aquila earthquake, Italy. Geology.

[32] Di Luccio F, Ventura G, Di Giovambattista R, Piscini A, Cinti FR (2010). Normal faults and thrusts reactivated by deep fluids: The 6 April 2009 M w 6.3 L’Aquila earthquake, central Italy. J. Geophys. Res..

[33] Stacey, F. D. *Physics of the Earth*. (John Wiley & Sons Inc, 1977).

[34] Montgomery DR, Greenberg HM, Smith DT (2003). Streamflow response to the Nisqually earthquake. Earth Planet. Sci. Lett..

[35] Doglioni C, Barba S, Carminati E, Riguzzi F (2014). Fault on–off versus coseismic fluids reaction. Geosci. Front..

[36] Terzaghi, K. Theoretical soil mechanics. *Géotechnique* 510, 10.1016/0167-1987(88)90005-0 (1943).

[37] Modoni G, Koseki J, Anh Dan LQ (2011). Cyclic stress–strain response of compacted gravel. Géotechnique.

[38] Boni CF, Bono P, Capelli G (1986). Schema Idrogeologico dell’Italia Centrale – A) Carta idrogeologica (scala 1:500.000); B) Carta idrologica (scala 1:500.000); C) Carta dei bilanci idrogeologici e delle risorse idriche sotterranee (scala 1:1.000.000). Mem. della Soc. Geol. Ital..

