# Supplementary material for: New insights into earthquake precursors from InSAR
Source: Sci Rep. 2017 Sep 20;7:12035. doi: 10.1038/s41598-017-12058-3 (PMC5607348; doi:10.1038/s41598-017-12058-3)
Supplement: Supplementary file 1 — Supplementary figures [file 41598_2017_12058_MOESM1_ESM.pdf]

Supplementary material for “New insights into earthquake precursors from InSAR”

Marco Moro[1*], **Michele Saroli**[2,1+], **Salvatore Stramondo**[1+], **Christian Bignami**[1+], **Matteo Albano**[1+], **Emanuela Falcucci**[1+], **Stefano Gori**[1+], **Carlo Doglioni**[1+], **Marco Polcari**[1+], **Marco Tallini**[3+], **Luca Macerola**[3+], **Fabrizio Novali**[4+], **Mario Costantini**[5+], **Fabio Malvarosa**[5+], **Urs Wegmüller**[6+]

[1] Istituto Nazionale di Geofisica e Vulcanologia, Via di Vigna Murata 605, 00143 Roma, Italy.

[2] Dipartimento di Ingegneria Civile e Meccanica (DICeM), Università degli Studi di Cassino e del Lazio meridionale, Via G. di Biasio 43, 03043 Cassino, Italy.

[3] Dipartimento di Ingegneria Civile, Edile-Architettura e Ambientale, Università dell'Aquila, Via Giovanni Gronchi 18, 67100 L’Aquila, Italy.

[4] TRE ALTAMIRA s.r.l., Ripa di Porta Ticinese, 20143, Milano, Italy.

[5] e-GEOS Via Tiburtina, 965, 00156, Rome, Italy.

[6] GAMMA Remote Sensing Research and Consulting AG, Worbstr. 225, CH-3073 Gümligen, Switzerland.

*marco.moro@ingv.it

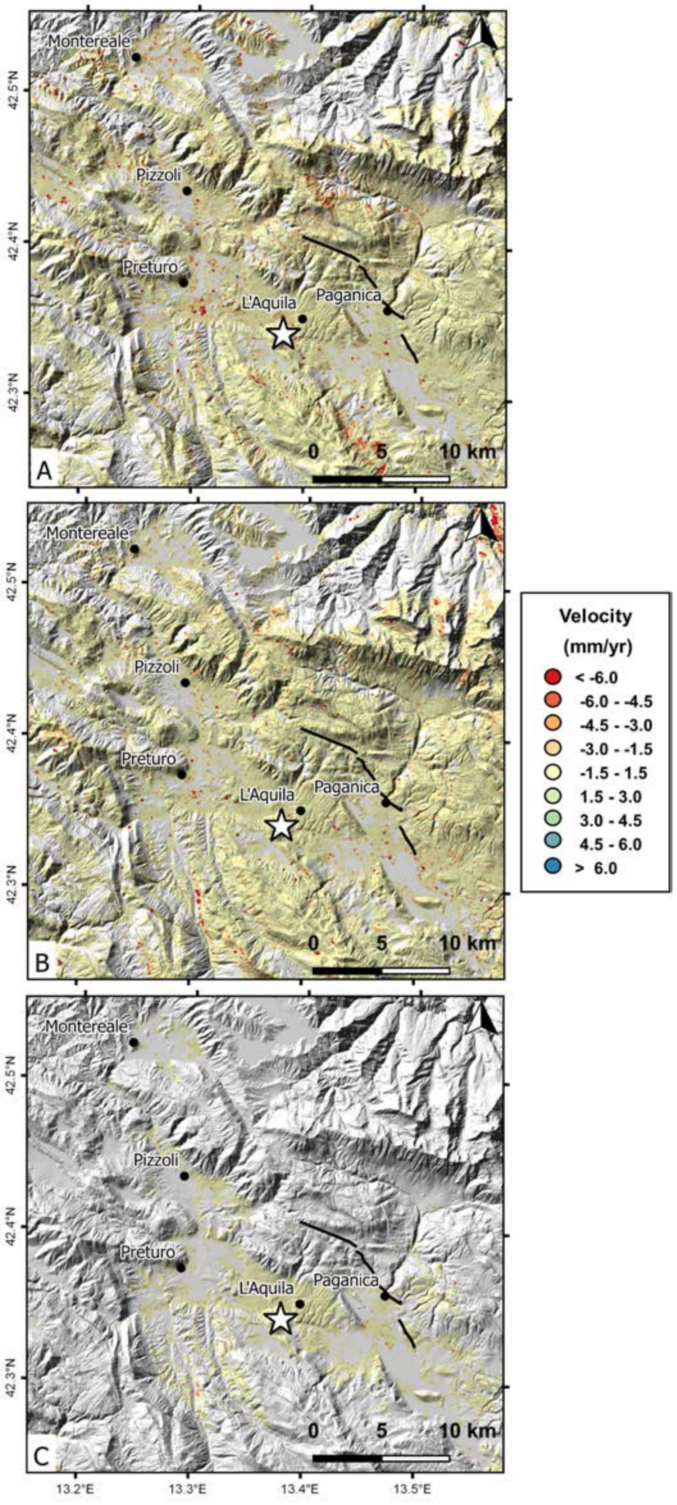

Figure S1. Maps of velocities extracted by performing multi-temporal InSAR processing of the RADARSAT-2 data acquired along ascending (A) and descending (B) orbits, and the ENVISAT data acquired along descending (C) orbits. The white star indicates the April 6, 2009 L'Aquila mainshock. The velocity maps do not show any significant pattern of deformation anywhere within the area. This figure was created with QGIS version 2.18.10 (QGIS Development Team, 2009. QGIS Geographic Information System. Open Source Geospatial Foundation. URL: http://qgis.osgeo.org).

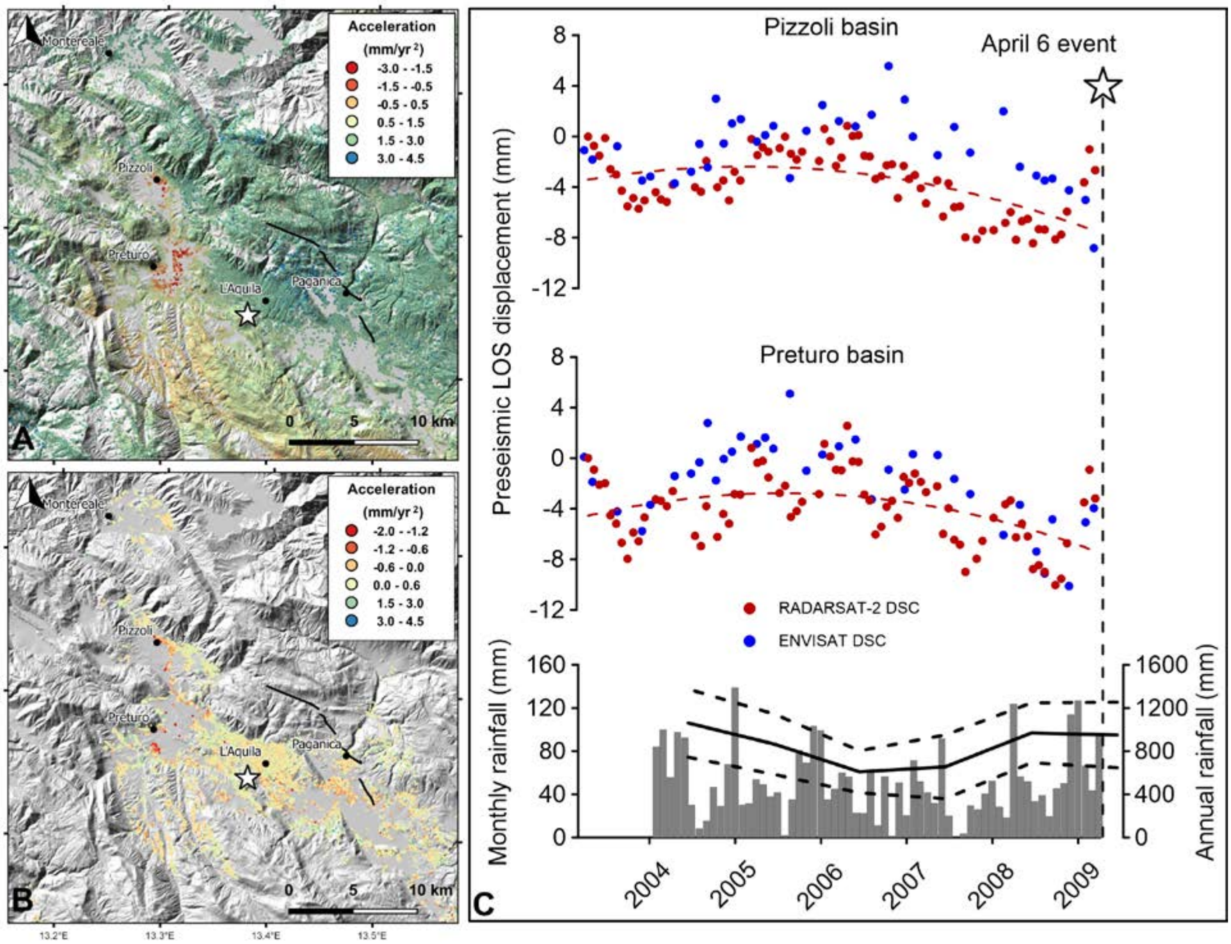

Figure S2. Maps of ground acceleration extracted by multi-temporal InSAR processing of the RADARSAT-2 (A) and ENVISAT (B) data acquired along a descending orbit. Although different sets of images and different techniques have been used, distinct patterns of negative values (red and orange persistent scatterers, PSs) are seen in both the RADARSAT-2 and ENVISAT acceleration maps within the Preturo and Pizzoli basins. This result confirms the consistency of the observed signal. The time series of the averaged PSs for the two basins (C) are shown with second-order polynomial fits (dashed lines) that emphasize the decelerating trends. The gray vertical bars indicate the monthly rainfall at the L'Aquila – S. Elia pluviometric station (the yellow triangles in panels A and B in Figure 1). The black line indicates the annual rainfall calculated as the mean value among eight pluviometric stations (see Figure 2 for the locations of the pluviometric stations). This figure was created with QGIS version 2.18.10 (QGIS Development Team, 2009. QGIS Geographic Information System. Open Source Geospatial Foundation. URL: http://qgis.osgeo.org) and Grapher® 8 from Golden Software, LLC (www.goldensoftware.com).

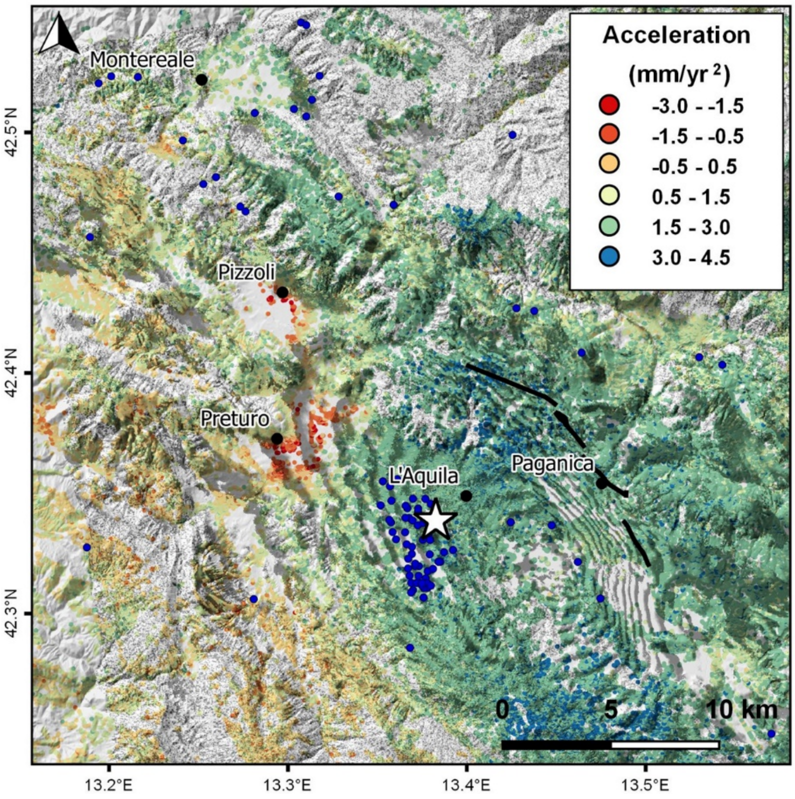

Figure S3. Ground acceleration map obtained using RADARSAT-1 data acquired along an ascending orbit superimposed on a coseismic interferogram obtained by applying differential InSAR to COSMO-SkyMed data. For completeness, the foreshocks (blue points) that occurred after January 2009 are shown on this map. Black lines represent the trace of the fault that caused the 2009 L'Aquila seismic event. This figure was created with QGIS version 2.18.10 (QGIS Development Team, 2009. QGIS Geographic Information System. Open Source Geospatial Foundation. URL: http://qgis.osgeo.org).

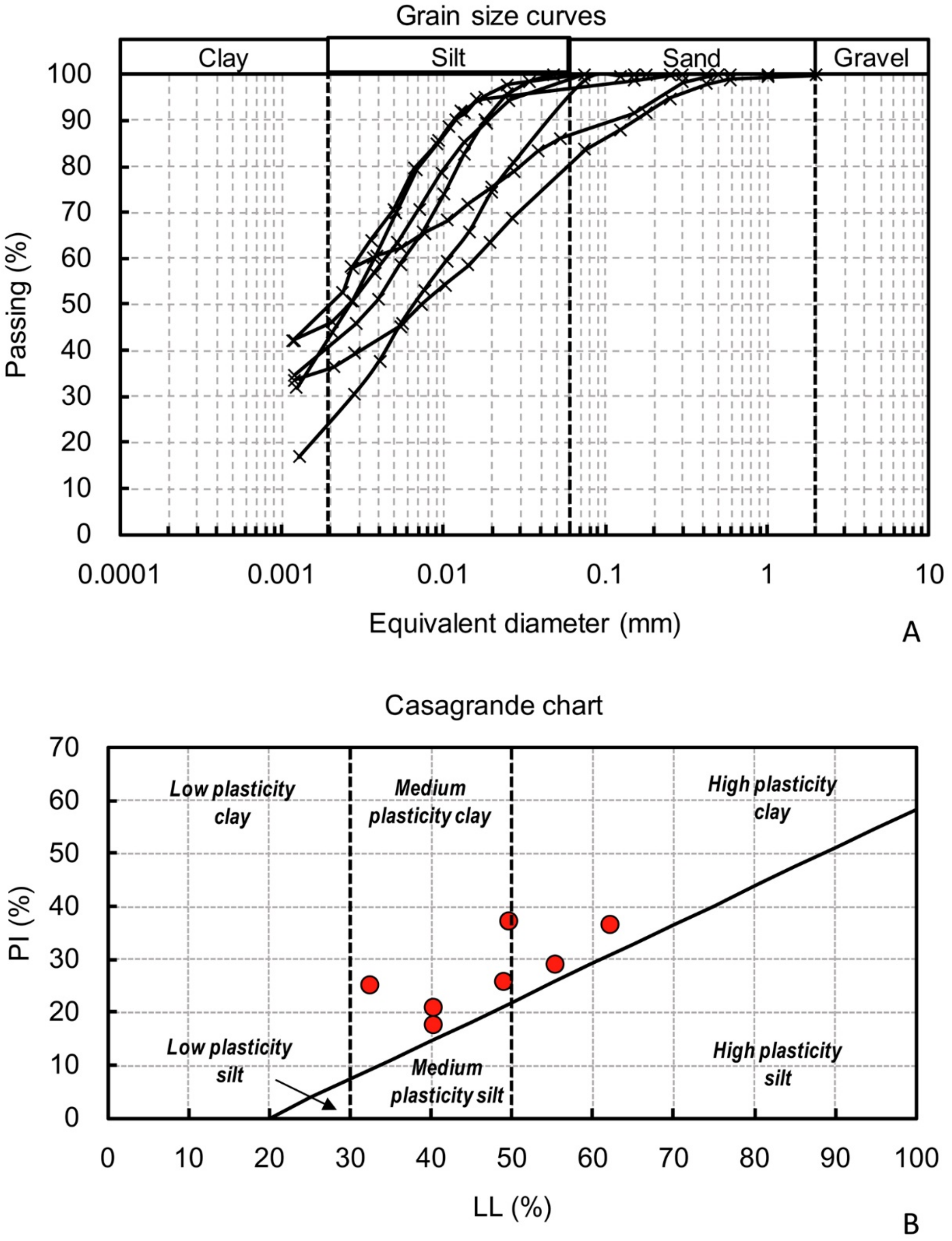

Figure S4. Geotechnical properties of the fine-grained soils filling the Preturo basin. (A) Grain size distributions obtained from seven specimens taken at different depths. (B) Casagrande chart. The abscissa shows the liquid limit (LL) index, whereas the ordinate shows the plasticity index (PI). This figure was created with Grapher® 8 from Golden Software, LLC (www.goldensoftware.com).

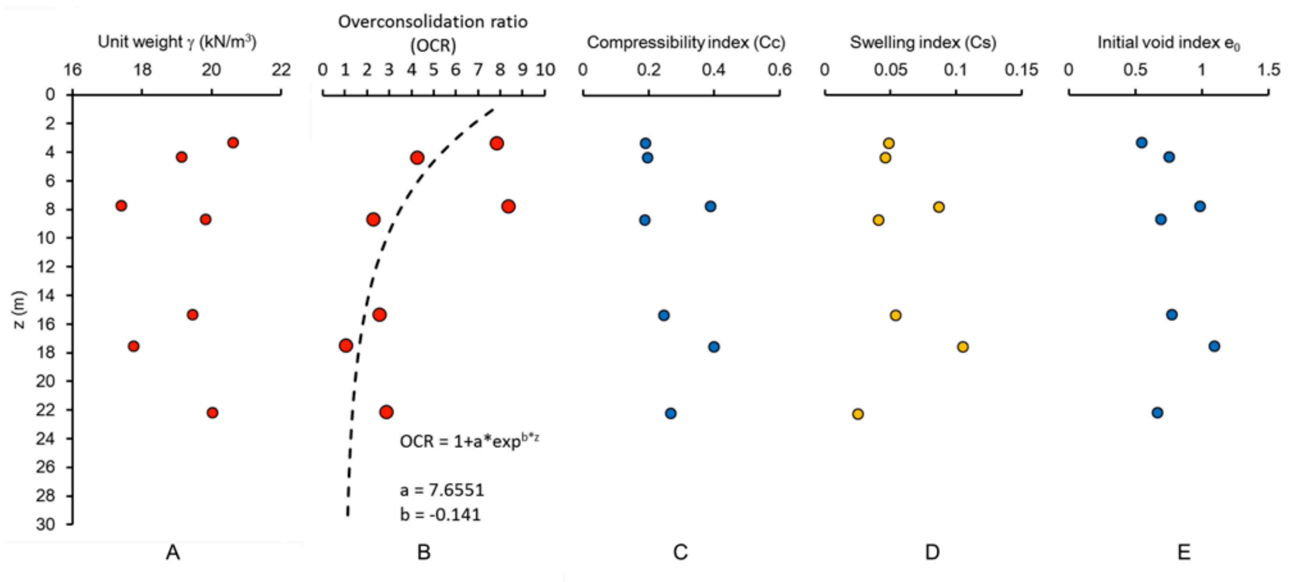

Figure S5. Experimentally determined values of the soil unit weight (A), the overconsolidation ratio (OCR) (B), the compressibility index (C_C) (C), the swelling index (C_S) (D) and the initial void index (e_0) (E) with depth. This figure was created with Grapher® 8 from Golden Software, LLC (www.goldensoftware.com).

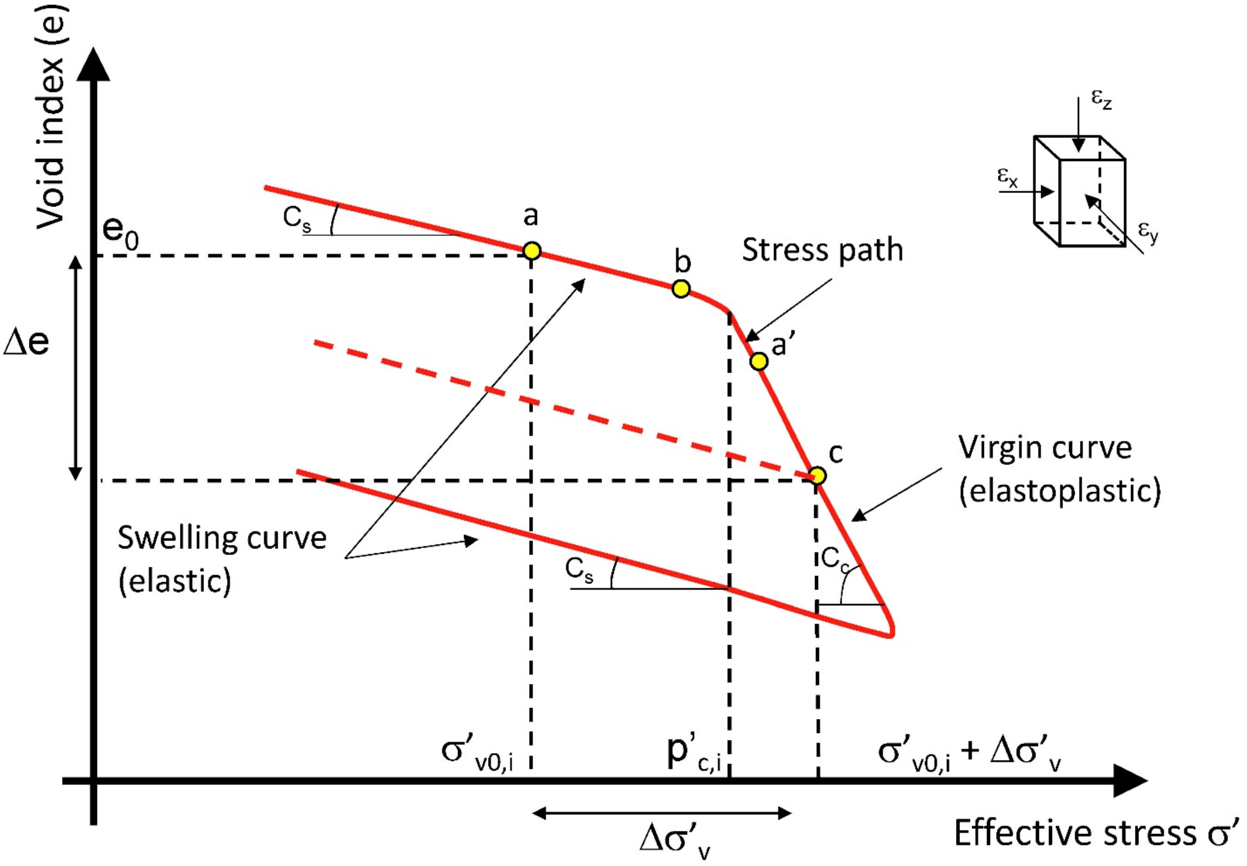

Figure S6. Typical stress path for a soil sample subjected to an increase and a decrease in the vertical stress under oedometric conditions ($\varepsilon_x = \varepsilon_y = 0$). This figure was created with Grapher® 8 from Golden Software, LLC (www.goldensoftware.com).
